# Contralateral Axon Sprouting but Not Ipsilateral Regeneration Is Responsible for Spontaneous Locomotor Recovery Post Spinal Cord Hemisection

**DOI:** 10.3389/fncel.2021.730348

**Published:** 2021-08-26

**Authors:** Yudong Cao, Ya Shi, Zhifeng Xiao, Xi Chen, Bing Chen, Bin Yang, Muya Shu, Yanyun Yin, Shuyu Wu, Wen Yin, Xianyong Fu, Jun Tan, Quanwei Zhou, Zhaoping Wu, Xingjun Jiang, Jianwu Dai

**Affiliations:** ^1^Department of Neurosurgery, Xiangya Hospital, Central South University (CSU), Changsha, China; ^2^State Key Laboratory of Molecular Developmental Biology, Institute of Genetics and Developmental Biology, Chinese Academy of Sciences, Beijing, China; ^3^Shigatse Branch, Xinqiao Hospital, Army Medical University (Third Military Medical University), Shigatse, China

**Keywords:** axon spouting, corticospinal tracts, neuronal regeneration, spinal cord hemisection, spontaneous locomotor recovery

## Abstract

Spinal cord injury (SCI) usually results in permanent functional impairment and is considered a worldwide medical problem. However, both motor and sensory functions can spontaneously recover to varying extents in humans and animals with incomplete SCI. This study observed a significant spontaneous hindlimb locomotor recovery in Sprague-Dawley rats at four weeks after post-right-side spinal cord hemisection at thoracic 8 (T8). To verify whether the above spontaneous recovery derives from the ipsilateral axonal or neuronal regeneration to reconnect the lesion site, we resected either the scar tissue or right side T7 spinal cord at five weeks post-T8 hemisected injury. The results showed that the spontaneously achieved right hindlimb locomotor function had little change after resection. Furthermore, when T7 left hemisection was performed five weeks after the initial injury, the spontaneously achieved right hindlimb locomotor function was dramatically abolished. A similar result could also be observed when T7 transection was performed after the initial hemisection. The results indicated that it might be the contralateral axonal remolding rather than the ipsilateral axonal or neuronal regeneration beyond the lesion site responsible for the spontaneous hindlimb locomotor recovery. The immunostaining analyses and corticospinal tracts (CSTs) tracing results confirmed this hypothesis. We detected no substantial neuronal and CST regeneration throughout the lesion site; however, significantly more CST fibers were observed to sprout from the contralateral side at the lumbar 4 (L4) spinal cord in the hemisection model rats than in intact ones. In conclusion, this study verified that contralateral CST sprouting, but not ipsilateral CST or neuronal regeneration, is primarily responsible for the spontaneous locomotor recovery in hemisection SCI rats.

## Introduction

Spinal cord injury (SCI) refers to devastating damage to the spinal cord tissue that results in a temporary or permanent irreversible impairment of sensory and motor function below the level of injury (Assinck et al., 2017). Clinically, a large percentage of patients with SCI are incompletely injured, with parts of spinal cord tissue remaining intact (Hollis et al., 2016). Although extensive spontaneous regeneration is rare in the adult mammalian central nervous system (CNS) after complete SCI, it has been recognized that significant spontaneous functional recovery commonly occurs after incomplete SCI in patients as well as animals (Raineteau and Schwab, 2001; Weidner et al., 2001; Maier et al., 2008; Spiess et al., 2009; Rosenzweig et al., 2010; Starkey et al., 2012; Hilton et al., 2016). At present, it is believed that CNS plasticity is associated with the recovery of locomotion after partial SCI (Weidner et al., 2001; Hagg, 2006; Rosenzweig et al., 2010; Seo and Jang, 2015). The plasticity includes various changes, ranging from alterations in the properties of reorganized neuronal circuits, injured axon regeneration, and collateral intact axon sprouting (Fouad and Tse, 2008; Onifer et al., 2011).

The corticospinal tract (CST) is the main descending motor pathway that controls skilled movements in the mammalian species; hence, spontaneous plasticity after SCI has mainly been studied for the corticospinal system (Rosenzweig et al., 2010). Several studies have shown that the formation of intraspinal detour pathways could mediate functional recovery after incomplete SCI (Bareyre et al., 2004; Courtine et al., 2008; van den Brand et al., 2012). Previous studies have found that the ipsilateral hindlimb CST axons amputated at the thoracic spinal cord level germinate new collateral branches in the cervical spinal cord. These collaterals then extend to the intermediate layers of the gray matter in the cervical spinal cord. There, they construct contacts with different groups of spinal interneurons, including long and short propriospinal neurons (Bareyre et al., 2004; Kerschensteiner et al., 2004; Jankowska and Edgley, 2006; Courtine et al., 2009; Lang et al., 2012). Nevertheless, the detailed mechanisms underlying spontaneous functional recovery are still somewhat speculative and unclear. In particular, the degree of recovery from the regeneration of severed axons versus compensation by spared axons is unclear. Moreover, in our recent study, we found that endogenous neurogenesis from activated spinal cord neural stem cells following complete acute SCI is achievable in both rodents and canines, and spontaneous locomotor recovery might result from new neuronal circuit reformation at the lesion site to reconnect the transected stumps (Li X. et al., 2017). Furthermore, functional scaffold implantation significantly increased the number of newborn neurons in the lesion site, which also resulted in neuronal regeneration and improved locomotor recovery (Li X. et al., 2017). At present, it is essential to uncover the primary mechanism pivotal for spontaneous locomotor function recovery after incomplete SCI: new neuronal circuits reorganized at the lesion site, the contralateral CST axons sprouting below the injury site, or direct axonal regeneration of the ipsilateral transected CST axons, to reconnect the original targets below the lesion site.

**FIGURE 1:**
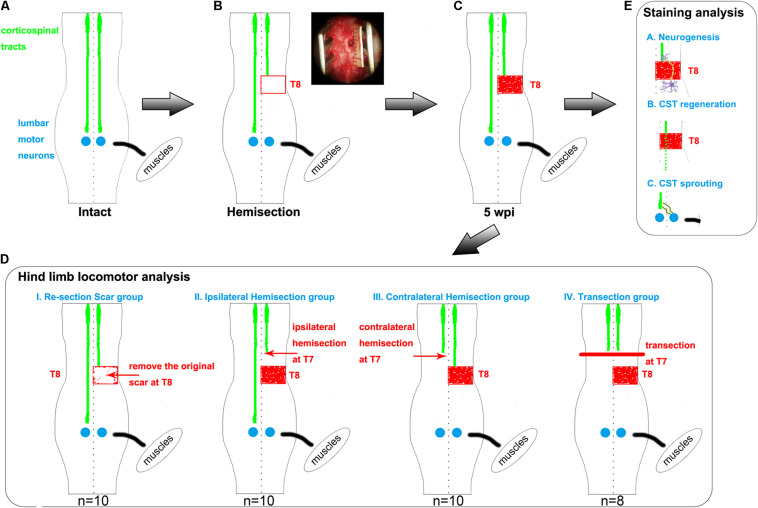
Hemisection model and different second operations for each group. **(A–C)** The establishment of hemisection model rats. **(A)** represents intact rats. **(B)** represents hemisection model rats that underwent a right hemisection at the T8 level. The red rectangle symbolizes the hemisection injury with a width of 2 mm. In the upper right corner, the surgery photo is taken under a microscope, visually displaying a full unilateral (right) hemisection operation. The red-filled rectangle in panel **(C)** indicates the scar observed five weeks post the hemisection injury. **(D)** Five weeks after the first hemisection operation at the T8 level, four different second operations are further established based on hemisection model rats. The red arrows indicate the second hemisection operation. The thick red line indicates a transection. The Re-section Scar group (*n* = 10) underwent a second hemisection operation at the original injury site to remove the scar. The Ipsilateral Hemisection group (*n* = 10) underwent a second hemisection operation on the ipsilateral side at T7. The Contralateral Hemisection group (*n* = 10) underwent a second hemisection operation on the contralateral side at T7. The Transection group (*n* = 8) underwent a transection operation at T7. **(E)** represents the possible mechanisms of the spontaneous locomotor recovery in hemisection model rats, including neurogenesis, CST axon regeneration, and CST axon sprouting. CST indicates the corticospinal tract.

This study constructed a rat spinal cord hemisection injury model by performing a full right side hemisection injury at thoracic 8 (T8, Figures 1A–C). At five weeks after the operation, spontaneous hindlimb locomotor recovery was evaluated. Rats with similar recovery levels were randomly divided into four groups to further investigate the related mechanism for the spontaneously achieved locomotor recovery. Rats in each group underwent one of four different surgeries: resection of the scar (T8), hemisection of T7 on the ipsilateral side, hemisection of T7 on the contralateral side, and complete transection of T7 (Figure 1D). By assessing the change in the achieved locomotor recovery of the right hindlimb among each group, we found contralateral CST axons sprouting, but not ipsilateral CST regeneration and neuronal circuit reformation, responsible for spontaneous locomotor recovery after spinal cord hemisection. Additionally, CST anterograde tracing and immunostaining results also validated the above conclusion (Figure 1E).

## Materials and Methods

### Ethics Statement

All animal procedures were performed in accordance with the National Institute of Health’s guidelines and were approved by the Animal Care and Use Committee of Xiangya Hospital, Central South University, Hunan Province, China. All efforts were made to ensure the welfare of the animals and to reduce suffering.

### Spinal Cord Hemisection Model

In this study, female Sprague-Dawley rats (weight 220–240 g) were housed on a 12 h light/dark cycle at 21°C. All rats were admitted to the new environment for one week before the beginning of any experimental procedures and provided free access to food and water throughout the experiment. All rats were deeply anesthetized with a mixture of ketamine hydrochloride and xylazine (80/5 mg/kg; ip). Postoperative care included butorphanol (0.1 mg/kg; sc) and ampicillin (100 mg/kg; ip) administration twice per day for 5 days. Bladders were expressed manually two or three times a day until voluntary control was resumed. After each surgery, the rats were placed in separate cages for seven days and then co-located with other rats. The rats in the experiment were acquired from the Vital River Laboratory Animal Technology Company.

To investigate the spontaneous recovery of motor function after incomplete SCI, we created a spinal cord hemisection model of rats that received a full unilateral (right) hemisection on the T8 spinal cord (Figures 1A,B). A 1.5 cm midline incision was made on the back skin to expose the T7-T9 vertebrae. Muscles were cut in layers along the midline, and laminectomy was performed at T8 using ophthalmic scissors. Lidocaine hydrochloride (5 mL: 0.1 g) was applied locally to the spinal cord to minimize pain and reduce stimulus during the surgery. Under the microscope, the dura mater was opened using the iris micro scissors and ophthalmic microforceps. The right hemisection of the spinal cord in T8 was then performed using a #11 scalpel blade, creating a lateral hemisection lesion with a width of 2 mm (Figure 1B). The muscle and skin layers were then closed using 4–0 silk sutures.

### Groups

To investigate whether newly formed neuronal circuits, sprouting axons, or regenerated axons contributed to the hindlimb locomotor recovery in the spinal cord hemisection model rats (Figure 1C), five weeks post T8 hemisection, rats with similar Basso, Beattie, Bresnahan (BBB) scores were randomly divided into four groups for a second surgery (Figure 1D). The first was the Re-section Scar group (*n* = 10), in which the second surgery was performed to remove the original scar in T8. The second was the Ipsilateral Hemisection group (*n* = 10), in which the second hemisection surgery was performed on the ipsilateral side at the T7 level. The third was the Contralateral Hemisection group (*n* = 10), in which the second hemisection surgery was conducted on the contralateral side at the T7 level. The fourth was the Transection group (*n* = 8), which received a complete transection at the T7 level.

### Anterograde Tracing of Corticospinal Tract

CST plays an essential role in the voluntary movement of the limbs. For visualizing the CST, some animals (*n* = 8), which were randomly chosen from the spinal cord hemisection model rats, were placed on a stereotaxic frame and received bilateral injections of the anterograde tracer biotinylated dextran amine (BDA, Dextran, Alexa Fluor^TM^ 594 or 488; 10,000 MW, Anionic, Fixable, 10% w/v solution in sterile saline) into the hindlimb motor cortex at a depth of 1.5 mm below the cortical surface according to the rat brain atlas (Büttnerennever, 1997; Chen et al., 2008; Fan et al., 2010). The injection sites’ stereotaxic coordinates were as follows: anteroposterior (AP) −1.0 mm, mediolateral (ML) ± 1.5 mm; AP −2.0 mm, ML ± 1.5 mm; AP −1.0 mm, ML ± 2.5 mm; AP −2.0 mm, ML ± 2.5 mm; AP −3.0 mm, ML ± 2.0 mm (0.5 μL per injection site for 10 min). Taking care to avoid damage to cortical surface vessels during the injection.

Four weeks after the BDA injections, the rats were perfused transcardially with 4% PFA, and the spinal cords were dissected, as mentioned above. After fixation and dehydration, the spinal cord specimens were sectioned into transverse and longitudinal sections penetrating the CST axons on a cryostat set at a thickness of 25 μm (Leica Microsystems GmbH, Germany). Images were captured using the Leica SCN400 slide scanner (Leica Microsystems, Germany). Besides, we performed unilateral CST antegrade tracing on intact rats (*n* = 4) for control.

### Immunofluorescence Staining

Five weeks after the first right hemisection in T8, seven hemisection model rats were perfused transcardially with 4% paraformaldehyde (PFA). The spinal cords were dissected, fixed overnight at 4°C, and shifted to 20% sucrose overnight at 4°C, followed by 30% sucrose (72 h at 4°C) until the specimens sank to the bottom (Li et al., 2016). Spinal cord lengths of 2 cm were cut, the center of which corresponded with the lesion site’s center. These 2 cm segments were then separately embedded in Tissue-Tek O.C.T. medium (Sakura Finetechnical Co., Japan) and cryosectioned at 10 μm thickness (Leica Microsystems GmbH, Germany). For immunofluorescence staining, the slides were incubated in phosphate-buffered saline (PBS) (containing 5% BSA and 0.1% Triton X-100) for 1 h at room temperature (RT). Then the slides were incubated with primary antibodies overnight at 4°C. The following primary antibodies were used: mouse monoclonal antibodies against neuronal specific nuclear protein (NeuN, 1:100, MAB377, Millipore) and microtubule-associated protein 2 (MAP2, 1:1,000, M1406, Sigma); rabbit polyclonal antibodies against glial fibrillary acidic protein (GFAP, 1:1,000, ab7779, Abcam). After incubating with primary antibody at 4°C overnight, the slices were taken out and washed with PBS three times, each time 5 min. Subsequently, the slides were incubated with Alexa Fluor 488-conjugated donkey anti-rabbit IgG (1:500, A21206, Invitrogen) and Alexa Fluor 594-conjugated donkey anti-mouse IgG (1:800, A21203, Invitrogen) for 1 h at RT. DAPI (1:1,000, Sigma) was used for cell nuclei staining. A Leica SCN400 slide scanner (Leica Microsystems, Germany) was used to capture these images.

### Behavioral Testing

We use the Basso-Beattie-Bresnahan (BBB) scores to evaluate the functional recovery of the right hindlimb one day after surgery and weekly thereafter (Basso et al., 1995, 1996, 2006). The hind limb movements of each animal were recorded using a camera and assessed offline by two independent observers blinded to the group details. Paw placement, joint movements, weight-bearing, and coordination among the limbs were used to evaluate the BBB locomotion scale.

### Image Analysis and Quantification

The histological images were analyzed using the NIH ImageJ analysis software by an investigator blinded to group assignments.

Contralesional CST axonal sprouting in response to spinal cord hemisection injury was detected at L3–L4 level. The intensity of the main BDA-labeled CST axons present in the contralesional funiculus was measured and averaged from 8 non-consecutive transverse sections randomly selected from each animal (*n* = 8 in hemisection model rats; *n* = 4 in intact rats) at L3–L4 level using Image J software in the Auto Threshold method. For BDA-labeled sprouting fibers analysis, the denervated gray matter was divided into three parts of the dorsal, intermediate, and ventral part using two horizontal parallel lines contiguous to the central gray matter border. The BDA labeling intensity within the denervated and intermediate gray matter (Figure 5B) was measured and averaged from the above 8 non-consecutive slides for each rat at L3–L4 level. To correct the inter-animal variation on the BDA labeling efficiency, we calculated the sprouting axon index as previously described (Lee et al., 2010; Soleman et al., 2012). Briefly, we used the quotient of the intensity of BDA-labeled CST sprouting axons in the denervated intermediate gray matter divided by the average intensity of the main BDA-labeled dorsal CST axons present in the contralesional dorsal funiculus for correction.

**FIGURE 2:**
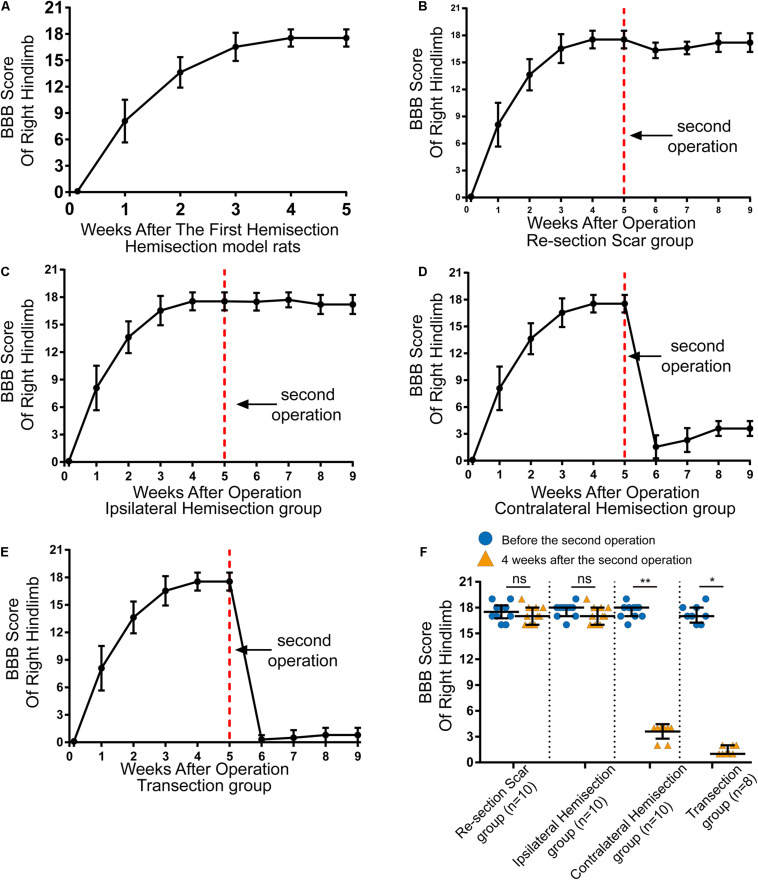
Locomotor assessment for hemisection model rats as well as locomotor changes after the second operation in four groups. **(A)** The BBB scores of the right hindlimb gradually improved spontaneously in hemisection model rats (*n* = 53). **(B–E)** Trends in BBB scores for each group based on the hemisection model rats: (B) Re-section Scar group (*n* = 10), **(C)** Ipsilateral Hemisection group (*n* = 10), **(D)** Contralateral Hemisection group (*n* = 10), **(E)** Transection group (*n* = 8). **(F)** Comparisons of BBB scores of the right hindlimb before the second operation and 4 weeks after that in each group. The red dotted line indicates the time point for the second operation in each group. *N* = 10 for Re-section Scar group, 10 for Ipsilateral Hemisection group, 10 for Contralateral Hemisection group, and 8 for Transection group. Bars indicate median, 25th-75th percentiles. *P < 0.05, **P < 0.01, data are presented as median ± IQR. BBB indicates Basso, Beattie, Bresnahan; ns, no sense; interquartile range, IQR.

**FIGURE 3:**
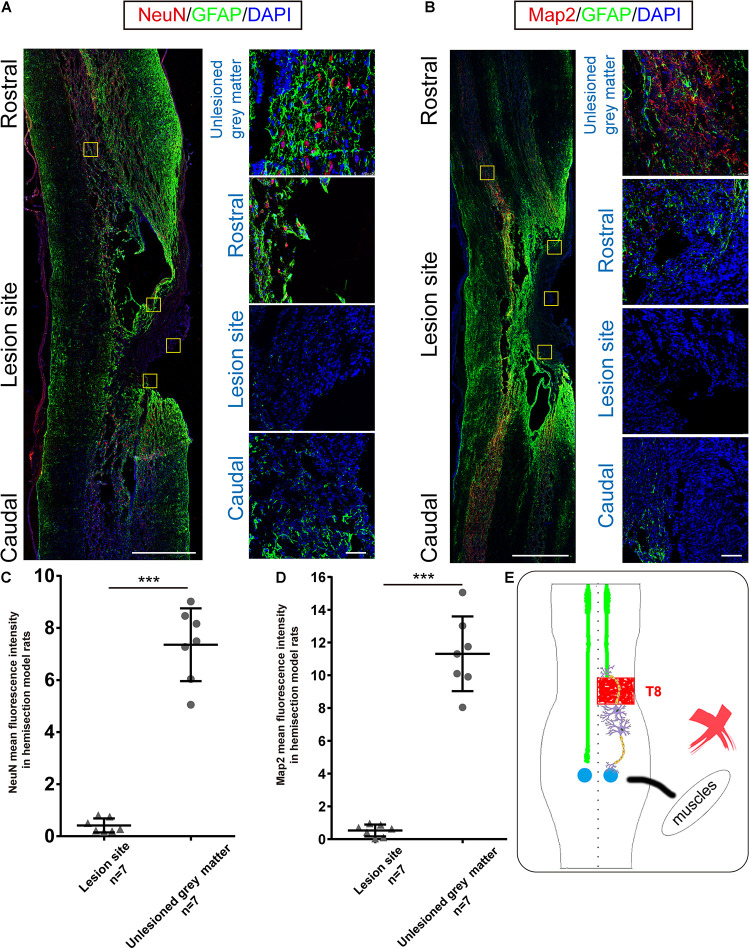
Immunohistochemical analysis in hemisection model rats. **(A,B)** Representative longitudinal sections of the spinal cord in hemisection model rats at five weeks. Four small squares on the right are local amplification of the corresponding site in the left longitudinal section. **(A)** NeuN, GFAP, and DAPI triple-staining. (red: NeuN, green: GFAP, blue: DAPI). **(B)** Map2, GFAP, and DAPI triple-staining. (red: Map2, green: GFAP, blue: DAPI). **(A,B)** exhibit the 2 mm defect caused by the first hemisection in hemisection model rats, and little Map2/NeuN positive cells are detected in the lesion area. Quantitative data **(C,D)** showed that the intensity of NeuN/Map2 positive signal in the unlesioned gray matter was significantly higher than in the lesion area in hemisection model rats. **(E)** The sketch map shows the neurogenesis contributing to the spontaneous locomotor recovery; however, this probability is excluded in the hemisection model rats. Bars indicate mean with SD. Data are presented as the mean ± SD. ***P < 0.001. Animal numbers = 7 for immunostaining. Scale bar: **(A,B)** left 1 mm; right 50 μm. NeuN indicates neuronal specific nuclear protein; Map2, microtubule-associated protein 2; GFAP, glial fibrillary acidic protein.

**FIGURE 4:**
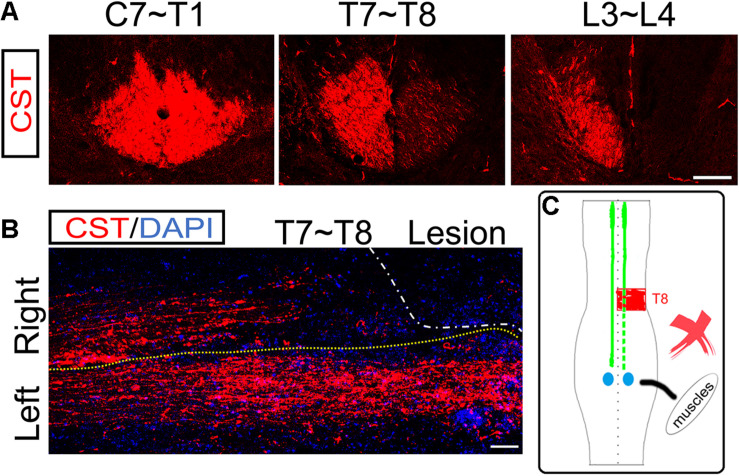
No ipsilesional CST axons regeneration across the lesion site. **(A)** Transverse sections of hemisection model rats show the bilateral CSTs are mainly located in the deep dorsal funiculus. **(B)** The representative longitudinal sections contiguous to those with the maximum diameter show that the contralesional rostral CST axons descend through the hemisection injury site and arrive at the caudal segment. However, the ipsilesional CST fibers were terminated in the hemisection injury site, indicating no CST axonal regeneration. **(C)** The sketch map shows the CST regeneration contributing to the spontaneous locomotor recovery; however, this probability is also excluded in the hemisection model rats. *n* = 8 for CST tracing in hemisection model rats. Scale bar, 50 μm. CST indicates the corticospinal tract.

**FIGURE 5:**
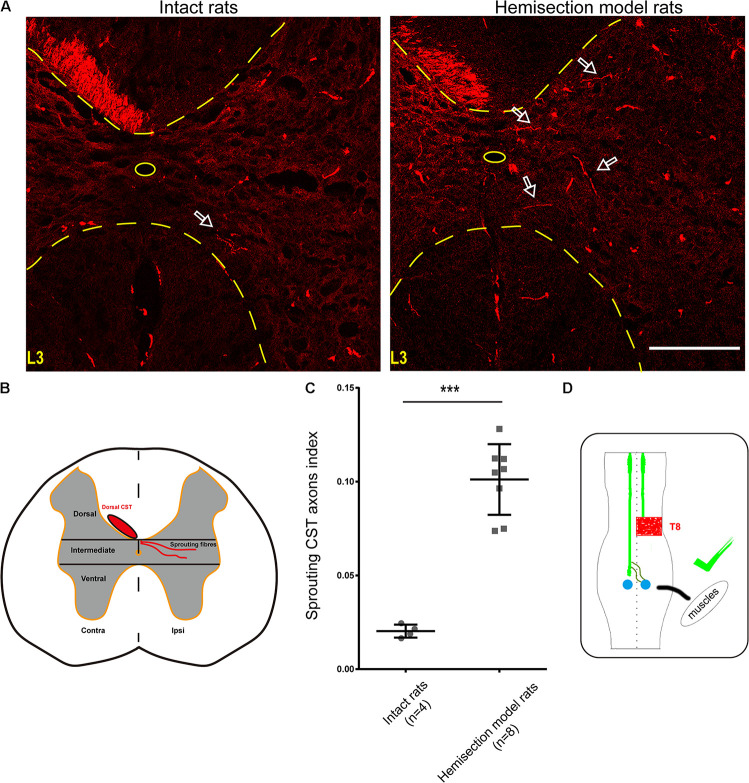
BDA-labeled contralesional CST fibers growing toward the ipsilesional denervated gray matter of lumbar spinal cord in the hemisection model rats. **(A)** Representative transverse sections at the lumbar enlargement level in the hemisection model and intact rats. The BDA-labeled CST in the contralesional side germinates collateral fibers into the denervated gray matter at L3 level in the hemisection model rats; in contrast, fewer CST fibers were detected to show collateral sprouting in intact rats. The arrows indicate the axons of the lumbar spinal cord in the hemisection model and the intact rats. **(B)** Lumbar cord gray matter is categorized into dorsal, intermediate, and ventral parts. **(C)** Quantified data show a significant increase in the index of CST sprouting axons in the denervated spinal cord after hemisection injury. **(D)** The sketch map shows the contralesional CST axon sprouting contributing to the spontaneous locomotor recovery, confirmed in the hemisection model rats. Bars indicate mean with SD. The data are presented as the mean ± SD. ***P < 0.001. *n* = 8 for CST tracing in hemisection model rats; *n* = 4 in intact rats. Scale bar, 150 μm. CST indicates the corticospinal tract; BDA, biotinylated dextran amine; CC, central canal.

To determine the expressions of Map2 or NeuN, 6 consecutive longitudinal spinal cord sections adjacent to the maximum diameter and centered on the hemisection site were selected from 7 hemisection model rats for measurement. The mean intensity of Map2- or NeuN positive signal in the intact gray matter was counted and averaged within three non-overlapping 40 × objective visual fields in each slice with ImageJ in the Auto Threshold method. The mean intensity of Map2- or NeuN positive signal in the hemisection lesion site were counted within three 40 × objective visual fields, respectively, from the rostral interface of the lesion, lesion center, and caudal interface of the lesion in each slice, and an average was obtained.

### Statistical Analysis

All statistical analyses were conducted using the software SPSS 19.0. All BBB scores results were presented as Median ± interquartile range (IQR). The scores data for preoperative and 4 weeks postoperative comparisons in each group were analyzed using a Wilcoxon matched paired signed-rank test for paired observations. The remaining data were represented by mean ± standard deviation (SD) except for the BBB score data. Comparisons of quantitative Immunofluorescence data and sprouting CST axons index in two groups were performed with a Two-tailed Student’s *t*-test. *P* values less than 0.05 were considered statistically significant.

## Results

### Spontaneous Locomotor Recovery of Right Hindlimb Post Spinal Cord Hemisection

In total, 59 female Sprague-Dawley rats underwent right side hemisection at T8, and finally, 6 rats were excluded from this study due to death or autophagy during and after surgery. On the first day after the hemisection surgery, none of the rats could move their right hindlimbs, and the BBB scores of the right hindlimb decreased to 0 ± 0 points (median ± IQR, Figure 2A). The locomotor function of the right hindlimb visibly improved in the 1st to 3rd week. The BBB scores continued to rise and reached a plateau (18 ± 1 points) by the 4th week (Figure 2A and Supplementary Video 1). Overall, even without any therapeutic intervention, the locomotor function of the right hindlimbs in these rats could be largely spontaneously restored over time after the hemisection injury.

### Locomotor Change Profiles After the Second Four Different Surgeries

Five weeks later, the above 38 hemisection model rats that spontaneously recovered their hind limb locomotor function to reach the plateau stage (18 ± 1 points on the BBB scale) were randomly divided into four groups and received the corresponding second operation as mentioned above (Figure 1D).

The results showed rats in the Re-section Scar (*n* = 10), and the Ipsilateral Hemisection (*n* = 10) groups exhibited little locomotor change in the right hindlimb after the second operation. The BBB scores of the Re-section Scar group dropped to 16 ± 0 points on the 1st day after the second operation. They were restored to 17 ± 2 points 4 weeks later (i.e., the ninth week after the first hemisection surgery, Figure 2B and Supplementary Video 2). Similarly, The BBB sores in the Ipsilateral Hemisection group have no noticeable decline and were 17 ± 2 points 4 weeks after the ipsilateral hemisection at T7 level (Figure 2C and Supplementary Video 3). There was no statistically significant difference in BBB scores before the second surgery and 4 weeks after that in the Re-section Scar and the Ipsilateral Hemisection groups (pre-second operation versus 4 weeks post-second operation, 17.5 ± 1.5 *vs.* 17.0 ± 2.0 in the Re-section Scar group, *P* = 0.429; 18.0 ± 1.0 *vs.* 17.0 ± 2.0 in the Ipsilateral Hemisection group, *P* = 0.393, Figure 2F).

However, in the Contralateral Hemisection and the Transection groups, the achieved movements of the right hindlimb were immediately abolished, with the BBB scores dropped to 0 ± 1 point after the second operation on the contralateral hemisection at T7 (*n* = 10) or T7 complete transection (*n* = 8). Although the hindlimb locomotor function began to restore overtime in the Contralateral Hemisection group, it improved only slightly with the BBB scores entering a platform level (4.0 ± 0.5 points) in the following 4 weeks (Figure 2D, Supplementary Video 4). Similarly, the BBB scores in the Transection group arrived at 1 ± 1 points 4 weeks after transection at the T7 level (Figure 2E and Supplementary Video 5). Notably, the BBB scores before the second surgery and four weeks after that showed significant differences in the Contralateral Hemisection and the Transection groups, respectively (pre-second operation versus 4 weeks post-second operation, 18.0 ± 1.0 *vs.* 4.0 ± 0.5 in the Contralateral Hemisection group, *P* = 0.005; 17.00 ± 1.75 *vs.* 1 ± 1 in the Transection group, *P* = 0.011, Figure 2F). These results implied that the contralesional hemispinal cord might play a vital role in the locomotor recovery after the unilateral SCI.

### No Neuronal Regeneration Was Detected in the Hemisection Site

Our previous BBB scores in the Re-section Scar and Ipsilateral Hemisection groups suggested that the ipsilesional hemispinal cord did not promote the spontaneous locomotion recovery after unilateral SCI. To further verify this argument, we examined the expressions of NeuN and Map2 in the hemisection model rats. Five weeks post spinal cord hemisection, immunofluorescence staining results of hemisection model rats (*n* = 7) showed intense staining of NeuN-positive cells mainly in the contralesional and ipsilesional intact gray matter; however, little NeuN-positive neurons could be detected throughout the lesion site (Figure 3A). The NeuN level in the undamaged gray matter (7.356 ± 1.397 AU) was significantly higher than that in the hemisection lesion area (0.416 ± 0.270 AU), and the difference between them was statistically significant (*P* = 0.000008, Figure 3C). The hemisection lesion site was atrophied and besieged by abundant GFAP-positive astrocytes (Figures 3A,B).

Similarly, Map2-positive cells were mainly distributed in the uninjured spinal tissue, and there was little Map2 + signal in and around the lesion site (Figure 3B). The level of Map2 in the undamaged gray matter (11.309 ± 2.283 AU) was higher than that in the lesion site (0.528 ± 0.360 AU), and the difference was also statistically significant (*P* = 0.000012, Figure 3D). The results above indicated no obvious neuronal circuit formed in the lesion site as a neuronal relay to re-bridge the transected stumps. The spontaneously achieved locomotor recovery after spinal cord hemisection did not result from neuronal regeneration throughout the lesion site (Figure 3E).

### No Ipsilateral CST Axon Was Regenerated to Cross the Lesion Site

To examine the role of ipsilesional CST axons in the spontaneous locomotor recovery after spinal cord hemisection, we performed BDA bilateral anterograde tracing to visualize the CST in the hemisection model rats (*n* = 8). The transverse sections of segments rostral to the hemisection site (C7 to T1 level) showed bilateral CSTs were situated in the dorsal funiculus (Figure 4A). Near the lesion site (T7 to T8 level), as many transected CST fibers were subjected to axonal degeneration, many BDA-labeled CST fibers were on the right hemispinal cord died back, and only some scattered staining signals could be detected (Figure 4A). The transverse sections of segments caudal to the lesion site (L3 to L4 level) only showed CST fibers on the left side, which indicated the precision of the hemisection model performed in this study (Figure 4A).

The longitudinal section showed that CST axons in the uninjured left hemispinal cord went through the T8 segment and entered into the lumbar spinal cord. However, the right CST axons stopped at T7 and could not pass through the injured site (T8 level) at five weeks post hemisection (Figure 4B). The above results showed that the ipsilesional transected CST fibers could not regenerate to cross the lesion area and reconnect the two stumps. So, there was not direct regeneration of CST axons into the lesion site that resulted in the spontaneous locomotor recovery after spinal cord hemisection (Figure 4C).

### Increased Contralesional CST Sprouting Was Observed at the Lumbar Enlargement

As mentioned above, we injected BDA into the bilateral cortices to track the CST in the hemisection model rats, and the ipsilesional CST signal disappeared in the rostral segment to the lesion site. To further investigate the relationship between the contralesional CST axons and spontaneous locomotion function recovery, we performed an additional unilateral CST tracing in four intact rats. In the transverse sections of intact rats at the lumbar enlargement (i.e., L3 to L4 level, animal numbers = 4), only a few BDA-labeled contralateral CST fibers crossed the midline into the ipsilateral gray matter (Figure 5A). Whereas hemisection injury obviously increased the number of BDA-labeled contralesional CST fibers sprouting into the denervated gray matter at the lumbar enlargement compared with the intact rats (Figure 5A). Then we quantified the labeled CST axon fibers crossing the midline by using the sprouting CST axons index. The sprouting CST axons index was four times in hemisection model rats (0.09277 ± 0.01774) than that measured in intact rats (0.02182 ± 0.00669), and there was a significant difference between these two groups (*P* = 0.000358, Figure 5C).

As many spinal interneurons in the lumbar enlargement indirectly control the hindlimb locomotor functions, these above results indicated that the sprouting CST fibers might have contacted the spinal interneurons, especially the propriospinal motor neurons, which further contributed to the spontaneous locomotor recovery of the hindlimb after spinal cord hemisection (Figure 5D). Meanwhile, these results were also consistent with the performance of the Contralateral Hemisection group, in which the achieved movement of the right hindlimb was immediately abolished.

## Discussion

In this study, we successfully established a full unilateral hemisection injury rat model at the T8 level. The injured rats could progressively and spontaneously restore the locomotor function of their related hindlimbs. Based on this model, by performing four different second operations, immunofluorescence staining, and CST tracing, we found that contralesional CST sprouting below the lesion segment contributes to locomotor recovery after unilateral SCI.

Ipsilesional hindlimbs was initially paralyzed after the first hemisection at the T8 level, but the contralesional hindlimb locomotion was less affected. The coordination between the forelimbs and hindlimbs on the ipsilesional side was also affected, possibly due to the interruption of various pathways after the unilateral SCI, which connect the cervical and lumbar segments and unite the forelimb and hindlimb locomotor (Helgren and Goldberger, 1993; Bem et al., 1995; Barriere et al., 2010). Over time, the locomotor function of the ipsilesional hindlimb gradually improved without any intervention, and four weeks later, the BBB scores reached a plateau of up to 18 ± 1 points. The representation of spontaneous recovery was consistent with other studies using this SCI model in rats (Ballermann and Fouad, 2006; Arvanian et al., 2009; Leszczyńska et al., 2015).

Many previous studies have shown that transplanting various engineered tissue materials or stem cells into the spinal cord hemisection area can improve the hindlimbs locomotor recovery (Fan et al., 2010; Wright et al., 2011; Jee et al., 2012; McCall et al., 2012; Estrada et al., 2014; Xu et al., 2017; Rosenzweig et al., 2018). Immunohistochemical studies also observed that the nerve fibers in the area rostral to the injured site increased and extended into the lesion site to innervate the denervated segment caudal to the lesion site, indicating nerve fibers regenerated and penetrated through the lesion site, thus promoting motor recovery after partial SCI (Fan et al., 2010; Wright et al., 2011; Jee et al., 2012; McCall et al., 2012; Estrada et al., 2014; Xu et al., 2017; Rosenzweig et al., 2018). However, another group drew contradictory conclusions. They performed two consecutive damages to the spinal cord of rats and pointed that the contralateral but not ipsilateral side played a vital role in the recovery process of locomotor function after incomplete SCI (Bareyre et al., 2004; Courtine et al., 2008; Li L. S. et al., 2017). Unfortunately, the above study did not implement a second hemisection at the lesion site (Courtine et al., 2008), so it could not be ruled out whether the axonal or neuronal regeneration in the lesion site led to the spontaneous locomotor recovery of the hindlimb after incomplete SCI. Therefore, in this study, we designed four different surgical approaches based on the spinal cord hemisection model to investigate whether ipsilesional regeneration or contralesional sprouting contributes to spontaneous motor recovery.

When the second hemisection operations were performed on the original scar (T8) or ipsilateral T7 level, it had almost no effect on the achieved hindlimb movements. However, when we performed contralateral hemisection or transection at the T7 level, the achieved movements vanished again. Therefore, it is not hard to speculate that the ipsilesional axonal or neuronal regeneration at the lesion site has little contribution to spontaneous locomotor recovery after the unilateral SCI. It is the contralesional spared axons that are very likely to be involved in the process of spontaneous recovery.

The longitudinal sections of hemisection model rats proved histologically that our hemisection surgery was pretty accurate. Some studies have shown that neuronal relays regenerated at the lesion site could restore hindlimb locomotor functions by reconnecting the two injured stumps in a complete SCI animal model (Yang et al., 2015; Fan et al., 2017; Li X. et al., 2017; Yin et al., 2018). However, our immunohistochemistry results showed almost no NeuN- and Map2-positive cells at the lesion site but many GFAP-positive cells around the lesion area. This immunostaining result suggested no newly generated neurons within the lesion site, which might rebuild a neuronal relay and thereby promote locomotor recovery.

Although many previous experiments have reported that the regeneration of axons, especially descending long tracts, such as the CST and reticulospinal tract, was the primary pathway to restore locomotor functions in a complete SCI model (Jee et al., 2012; McCall et al., 2012; Lewandowski and Steward, 2014; Rodriguez et al., 2014; Ruschel et al., 2015), in the present study, we were unable to detect the regeneration of ipsilesional CST fibers across the lesion site in the hemisection model rats. Furthermore, we detected significant contralesional CST axon fibers sprouting into the ipsilesional denervated gray matter at the lumbar enlargement. In contrast, we performed unilateral CST tracing by BDA in intact rats but found no obvious CST axon sprouting into the contralateral gray matter. Accordingly, the contralesional axon sprouting into the ipsilesional gray matter might explain why these rats showed significant spontaneous locomotor recovery in the ipsilesional hindlimb after unilateral SCI; however, no neuronal and CST axonal regeneration within the hemisection site was observed.

This study also showed that a gradual spontaneous recovery in a meager degree appeared in the Transection and Contralateral Hemisection groups, even if the segment caudal to the spinal cord lesion lost any connections to the brain. This phenomenon is consistent with the findings of other researchers (Tillakaratne et al., 2010). At present, this is primarily attributed to the local activities of central pattern generators (CPGs), which are present in many species (Grillner, 1985; Deliagina et al., 1999; Rossignol et al., 2007; Etlin et al., 2010); CPGs of rats are located in the lumbar enlargement (Magnuson et al., 1999; Zhang et al., 2017). Therefore, we argue that the contralesional CST and other descending axon sprouting into the denervated gray matter are the main but not the only factor in promoting spontaneous locomotor recovery after unilateral SCI in rats.

Our studies assessed spontaneous recovery from unilateral SCI and verified the importance of axon reorganization for functional recovery without applying any treatment intervention. However, the CST is not the only descending tract that controls movement. Some studies have shown that spared reticulospinal fibers might be involved in locomotion recovery following incomplete spinal cord injury through spontaneous compensatory sprouting below the lesion site (Ballermann and Fouad, 2006). Therefore, other descending motor tracts may also be associated with spontaneous locomotor recovery after unilateral SCI. On the other hand, the molecular regulation of this reorganization’s different steps is much less understood. A deeper understanding of these plastic mechanisms will promote the development of new rehabilitative methods for paralyzed patients. Further research is needed to confirm whether similar results can be observed when therapeutic interventions are used within the same experimental injury mode and further investigate the molecular signals of the plasticity of uninjured axons.

In conclusion, rats can spontaneously and significantly restore locomotor function after unilateral SCI at the thoracic level. The contralesional uninjured spinal cord plays a crucial role in this spontaneous recovery. The possible mechanism underlying this is that the contralesional spared CST axons sprout, extend toward the denervated gray matter, promote the synaptic formation, thereby contribute to locomotor function recovery.

## Data Availability Statement

The original contributions presented in the study are included in the article/Supplementary Material, further inquiries can be directed to the corresponding author/s.

## Ethics Statement

The animal study was reviewed and approved by the Animal Care and Use Committee of Xiangya Hospital, Central South University, Hunan Province, China.

## Author Contributions

YC, XJ, and JD conceived and designed the study. YC performed the experiment and wrote the manuscript. YS, ZX, XC, BC, BY, MS, and YY contributed reagents and analyzed the results. YC, SW, WY, XF, JT, QZ, and ZW performed the image visualization. All authors contributed to the article and approved the submitted version.

## Conflict of Interest

The authors declare that the research was conducted in the absence of any commercial or financial relationships that could be construed as a potential conflict of interest.

## Publisher’s Note

All claims expressed in this article are solely those of the authors and do not necessarily represent those of their affiliated organizations, or those of the publisher, the editors and the reviewers. Any product that may be evaluated in this article, or claim that may be made by its manufacturer, is not guaranteed or endorsed by the publisher.

## Abbreviations

SCI: spinal cord injury
CST: corticospinal tract
CNS: central nervous system
BBB: Basso, Beattie, Bresnahan
BDA: biotinylated dextran amine
AP: anteroposterior
ML: mediolateral
PFA: paraformaldehyde
PBS: phosphate-buffered saline
RT: room temperature
NeuN: neuronal specific nuclear protein
MAP2: microtubule-associated protein 2
GFAP: glial fibrillary acidic protein
IQR: interquartile range
SD: standard deviations
CPGs: central pattern generators.
